# Environmental Approaches to Prevention in College Settings

**Published:** 2011

**Authors:** Robert F. Saltz

**Keywords:** Alcohol consumption, harmful drinking, college students, prevention, intervention, environmental-level intervention, community-level intervention, individual-level intervention, prevention program, A Matter of Degree, Southwest DUI Enforcement Project, Neighborhoods Engaging With Students, Study to Prevent Alcohol-Related Consequences, Safer California Universities

## Abstract

Because of concerns regarding drinking among college students and its harmful consequences, numerous prevention efforts have been targeted to this population. These include individual-level and community-level interventions, as well as other measures (e.g., online approaches). Community-level interventions whose effects have been evaluated in college populations include programs that were developed for the community at large as well as programs aimed specifically at college students, such as A Matter of Degree, the Southwest DUI Enforcement Project, Neighborhoods Engaging With Students, the Study to Prevent Alcohol-Related Consequences, and Safer California Universities. Evaluations of these programs have found evidence of their effectiveness in reducing college drinking and related consequences. The most effective approaches to reducing alcohol consumption among college students likely will blend individual-, group-, campus-, and community-level prevention components.

Because of the concerns regarding drinking among college students and its harmful consequences to the students, their families, communities, and society as a whole, the identification of prevention strategies shown to be effective in college populations was a fundamental objective of the National Institute on Alcohol Abuse and Alcoholism’s (NIAAA) Task Force on College Student Drinking (NIAAA 2002). In its conclusions and recommendations, the Task Force categorized available interventions into four tiers, based on the level of empirical support found in the research literature. Tier 1 strategies have the strongest evidence of efficacy; they include such approaches as brief interventions, cognitive–behavioral interventions, and expectancy challenge, all of which are aimed at individual students. By identifying these strategies, the Task Force provided a valuable service to both the college and research communities, and with enhanced funding from NIAAA, additional research on these programs and strategies has continued in the years since (for more information, see the article by Cronce and Larimer, pp. 204–221).

The Task Force also drew attention to other prevention strategies reported in the research literature that had been shown to be effective in general populations but for which essentially no evidence of efficacy for colleges and universities existed. These Tier 2 strategies included such universal alcohol control measures as enforcing laws related to the minimum drinking age and reducing alcohol-impaired driving, raising the price of alcoholic beverages, reducing the density of alcohol outlets, and promoting responsible beverage service among retailers. Because these interventions typically require action at the community level, the Task Force stated that “[the] formation of a campus and community coalition involving all major stakeholders may be critical to implement these strategies effectively” (NIAAA 2002, p. 20). In calling for research on these Tier 2 strategies in college communities, the Task Force report prompts the reasonable question of why additional interventions should be studied. Community-based prevention interventions would seem difficult to design and implement and even more difficult to evaluate. For example, when an entire campus or community is the unit of intervention, a rigorous research design would require multiple intervention or control conditions as well comparison campuses, preferably with random assignment to intervention condition. With this level of effort required, it is little wonder that such studies are rare. Why, then, would it not be sufficient to just further develop and improve the existing individual-level Tier 1 interventions?

Several reasons support additional attention to community-level Tier 2 interventions as well as to the Tier 1 interventions. First, in their current form, many of the Tier 1 interventions are labor intensive and require skilled people to conduct them, even if there are promising efforts to overcome these potential barriers. If these interventions were to be adopted for all students, they would require time for screening each student, plus the time needed to deliver brief interventions for those who screen positive. Thus, adopting these strategies as campus-wide efforts would result in many research, cost, recruitment, and logistical challenges.

Second, the Tier 1 interventions are most appropriate for students whose drinking already is problematic or who at least are members of subgroups who drink more heavily than the general population (see Larimer and Cronce 2002, 2007). However, alcohol-related harm is not limited to those whose drinking can be characterized as consistently heavy or risky (Gruenewald et al. 2003; Weitzman and Nelson 2004). At the population level, light and moderate drinkers outnumber the heaviest drinkers to such an extent that, even though they have a lower level of individual risk, they are responsible for the majority of alcohol-related problems (see Kreitman 1986). Therefore, interventions aimed at risky drinkers should be complemented by universal prevention strategies.

Third, it is possible that ignoring the broader campus/ community environment actually may reduce the impact of otherwise effective individually targeted interventions. For example, DeJong and colleagues (2009) tried to replicate a social-norms marketing campaign that aimed to correct students’ overestimation of peer drinking. When they failed to replicate the original positive effects of the intervention, the investigators concluded that the intervention was thwarted at campuses surrounded by a high density of alcohol outlets. Thus, the intervention seemed to be unable to overcome the environmental risk produced by the number of places to buy alcohol

Fourth, limiting recommendations to college student-specific interventions alone would ignore the progress that has been made over the past decades in identifying effective universal prevention strategies that reasonably could be expected to work in college campus and community settings. Finally, there is every reason to believe that the greatest chance of creating safer college campuses will derive from a strategic combination of individual-, group-, campus-, and community-level interventions to form a holistic approach that maximizes positive effects through a synergistic effect.

This article provides an overview of some of the general-population and college-specific environmental interventions that have been studied and implemented and their role in reducing the risks and harmful consequences associated with college drinking.

## General-Population Environmental Interventions

A primary focus of the NIAAA Task Force was a summary review by Hingson and Howland (2002), and later updated by Hingson and colleagues (2009), of comprehensive community interventions that impacted college settings and included a variety of health outcomes beyond alcohol-related outcomes. Among the wide variety of interventions described by those authors, three examples of community interventions were closely related to the research on college-specific interventions: the Massachusetts Saving Lives program, the Communities Mobilizing for Change on Alcohol (CMCA) program, and the Community Trials Project.

The Massachusetts Saving Lives program (Hingson et al. 1996) targets drunk driving and speeding through activities such as drunk-driving checkpoints, speed-watch telephone hotlines, police training, alcohol-free prom nights, beer-keg registration, business information campaigns, media campaigns, and increased surveillance of attempts by minors to buy alcohol. The program also paid a great deal of attention to media advocacy, seeking to create and shape news stories in ways to support the prevention efforts. After the implementation of the program, self-reported driving after drinking among those under age 20 dropped from 19 percent to 9 percent, the prevalence of speeding was cut by 50 percent, and alcohol-related traffic deaths were reduced 45 percent more in the treatment cities compared with the rest of the State over the project’s 5-year period (Hingson et al. 1996).

The CMCA program focused on alcohol availability to youth in seven small to mid-sized communities in Minnesota and Wisconsin, with another eight communities serving as comparisons. With this program, an organizer working with each community mobilized support for a variety of activities, including increased enforcement of laws prohibiting alcohol sales to youth as well as enhanced awareness of the problem and importance of enforcement to the community at large. As a result, alcohol sales to underage decoys were reduced in the intervention communities, and surveys of youth showed a decline in attempts to purchase alcohol, provide alcohol to peers, and consume alcohol (Wagenaar et al. 2000*a*,*b*). Drunk-driving violations also were reduced in the intervention communities. Of interest, the program seemed to have the greatest effect on the oldest underage individuals—that is, on those who were of traditional college age—even though the study was not targeted specifically to college students.

The Community Trials Project targeted alcohol-related injuries and deaths in three communities (each with a matched community for comparison). Specific components included responsible beverage-service training and enforcement, increased enforcement of drunk-driving laws (and public perception of that increase), enforcement of underage sales laws, reduced alcohol availability via curtailing of outlet density, and mobilization of the community and its leaders in support of these interventions. The intervention reduced alcohol-involved crashes by more than 10 percent over the comparison communities and reduced alcohol-related assaults by over 40 percent (Holder et al. 2000).

## College-Specific Environmental Interventions

Since the Task Force report was issued, a few studies of multicomponent community-based college interventions have been reported in the literature but, as summarized in an update by Toomey and colleagues (2007), most of them used weak study designs (e.g., no comparison campuses). The following paragraphs describe some of programs that have been evaluated in recent year.

### A Matter of Degree

One of the studies with a good design was an evaluation of the American Medical Association’s A Matter of Degree program. Weitzman and colleagues (2004) compared this comprehensive environmental community intervention comprising such strategies as reduced alcohol availability, enhanced enforcement of serving laws, and restrictions on alcohol advertising that was implemented at 10 schools with a high prevalence of heavy drinking with 32 similar campuses that did not receive the intervention. The investigators first found no significant differences in level of drinking between the intervention and comparison schools. However, when they compared a subset of five campuses that implemented the program with greater intensity with the comparison schools, they found significantly lower rates of heavy drinking and alcohol-related negative consequences at the intervention schools (Weitzman et al. 2004).

### Southwest DUI Enforcement Project

Clapp and colleagues (2005) evaluated a driving-under-the-influence (DUI) prevention program on one college campus. The program included enhanced enforcement via roadside checkpoints and patrols, accompanied by a media advocacy campaign and a social marketing effort. At the intervention campus, self-reported DUI decreased (odds ratio of .55), whereas no change was reported among students at a comparison school. As the investigators noted, the study was limited because it did not use a more rigorous design involving random assignment and multiple campuses. Nevertheless, the results are suggestive of what might be possible in an enhanced replication.

### Neighborhoods Engaging With Students

The Neighborhoods Engaging with Students (NEST) project is an example of a multicomponent community intervention that was developed and implemented at Western Washington University (WWU). The program comprised a combination of alcohol-control measures and an education campaign. Enforcement interventions included increased patrols looking out for parties and/or alcohol as well as increased compliance checks at on-premise and off-premise establishments within 2 miles of the campus. These measures were supplemented by student-targeted publicity, such as advertisements in the student newspaper and articles in the local media. In addition, neighborhood engagement interventions focused on educating students regarding the rights and responsibilities associated with living in that community and sought to integrate students into neighborhood organizations and activities. Moreover, students who received minor-in-possession-of-alcohol citations were required to complete community service in those neighborhoods through a program called the Neighborhood Service Alternative Project. Finally, there was an increase in late-night programming on campus that focused on first-year students. An evaluation of the implementation of this project was conducted using three public universities in Washington in 2005 and 2006: While WWU implemented the NEST program, a second university was funded to implement a very similar program, and a third campus served as a comparison site. Analysis via hierarchical linear modeling demonstrated a reduction in heavy episodic drinking in both intervention schools (odds ratio of .73) compared with the school with no intervention (Saltz et al. 2009).

### The Study to Prevent Alcohol-Related Consequences

The Study to Prevent Alcohol-Related Consequences (SPARC) is a comprehensive intervention using a community-organizing approach to implement environmental strategies in and around college campuses (Wolfson et al. 2007, in press) with the ultimate goal of reducing high-risk drinking and alcohol-related consequences among college students. Eight public and two private universities in North Carolina were randomized to the intervention or a comparison condition (i.e., no intervention). A repeated cross-sectional design was used to assess impact of the intervention. Each intervention school was assigned a campus/community organizer who worked to form a campus-community coalition that developed a unique strategic plan, which then was implemented over a period of 3 years. Although each campus was able to develop its own prevention plan, all campuses were required to choose three of four general strategy domains—that is, reduce alcohol availability, address price and marketing of alcoholic beverages, improve social norms, and minimize harm related to alcohol. Within the selected general category, the interventions were expected to be comprehensive, comprising policy, enforcement, and awareness. Examples of specific components included social-norms marketing to correct students’ misperception of peer drinking, restricting alcohol at campus events, and enforcing compliance to laws prohibiting sales to underage people.

Wolfson and colleagues (2007, in press) found decreases in the intervention group compared with the control group both in severe consequences resulting from the students’ own drinking and in alcohol-related injuries caused to others. Thus, students on the intervention campuses who experienced severe consequences as a result of their own drinking dropped from 18 percent to 16 percent; likewise, alcohol-related injuries caused to others dropped from 3.5 percent to 2.0 percent of students at the intervention campuses. At the population level, these reductions translate to approximately 200 fewer severe consequences per month on each campus and 100 fewer injuries caused to others per month, representing a very meaningful impact. Additional analyses demonstrated that higher levels of implementation of the intervention were further associated with reductions in interpersonal consequences resulting from others’ drinking and alcohol-related injuries caused to others.

### Safer California Universities

The Safer California Universities study was designed to test the efficacy of a community-based environmental alcohol risk management strategy applied to college campuses (Saltz 2010). The intervention included nuisance party enforcement operations (i.e., “party patrols”), minor decoy operations, DUI checkpoints, social-host ordinances, and the use of campus and local media to increase the visibility of these strategies. The investigators then used a controlled, randomized experimental design involving 14 public universities, one-half of which were randomly assigned to the intervention condition and the other one-half served as comparison campuses. Annual surveys of randomly selected undergraduate students assessed the student’s drinking behavior in six different settings during the fall semester—that is, at residence-hall parties; campus events; fraternity or sorority parties; and parties at off-campus apartments or houses, in bars or restaurants, or in outdoor settings. The study specifically measured the proportion of drinking occasions during which students drank to intoxication in these settings, the proportion of students who reported any intoxication at each setting during the semester, and whether students drank to intoxication the last time they went to each setting.

Significant reductions in the incidence and likelihood of intoxication at off-campus parties and in bars and restaurants were observed for the intervention universities compared with the control universities. Moreover, students at the intervention universities also had a lower likelihood of intoxication the last time they drank at an off-campus party (odds ratio 0.81), a bar or restaurant (0.76), or across all settings (0.80) (Saltz 2010). The magnitude of these effects translated to approximately 6,000 fewer cases of intoxication at off-campus parties per semester at each campus and 4,000 fewer cases of intoxication at off-campus bars and restaurants. Nearly as important was the finding that no increase in intoxication (i.e., displacement) appeared in other settings. Furthermore, stronger intervention effects were achieved at the intervention universities with the highest intensity of implementation.

## Other Campus Prevention Interventions

The NIAAA Task Force also cited a host of other policies, programs, and strategies that have been offered for prevention on college campuses, although most of these measures have not been specifically evaluated. These measures were designated as Tier 3 strategies—that is, interventions that seemed logical or promising and good candidates for evaluation. These included such interventions as holding Friday classes and exams to reduce Thursday night partying, expanding alcohol-free late-night student activities, establishing alcohol-free dormitories, controlling or eliminating alcohol at sports events, refusing sponsorship gifts from the alcohol industry to avoid perceptions that underage drinking is acceptable, and banning alcohol on campus, including at faculty and alumni events (NIAAA 2002). In addition, many researchers and practitioners point to the need for event-specific prevention strategies that can be relevant to college populations. These seek to address student drinking associated with peak times and events, such as orientation and beginning of the academic year, 21st birthday celebrations, spring break, and graduation (Neighbors et al. 2007). Toomey and colleagues (2007) provide additional examples of such approaches in their review of specific campus-level strategies.

Evidence also exists about strategies that are not effective, labeled Tier 4 strategies. These chiefly include educational interventions that only provide information about alcohol and alcohol-related harms. Although education often is one element of effective multimodal interventions, educational programs in isolation repeatedly have been shown to be ineffective. Nevertheless they often are favored by institutions because they are inexpensive, easy to implement, noncontroversial, and fit well in institutions of higher learning that are characterized by great faith in the efficacy of information per se. Other strategies, such as using breath-analysis tests to give students information on their blood alcohol concentration (BAC) or “designated driver” schemes, also have been shown to be ineffective in reducing drinking, and the former even may encourage competition for achieving the highest BAC (see the discussion of Tier 4 strategies in NIAAA [2002]).

Recently, however, a new generation of online, electronic educational interventions has appeared that bear a resemblance to information strategies but incorporate features found in effective cognitive–behavioral or brief motivational individual interventions. Specific programs that have been evaluated in recent years include myStudentBody, CollegeAlc, Alcohol eCheckup to Go (e-Chug), and AlcoholEdu. Of note, they all incorporate personalized feedback based on the data the student enters on his or her drinking behavior. The students also are shown how their own drinking compares to that of their peers. These programs typically incorporate interactive components along with information about alcohol and its effects, and some also provide students with tips or skills for monitoring and limiting their drinking. For more information on such programs, see the sidebar in this issue by Walters and Neighbors, p. 222.

In a recent evaluation of the e-Chug and AlcoholEdu programs, Hustad and colleagues (2010) provide a concise summary of the work done in this area. However, given the small number of evaluations and their methodological weaknesses, it is premature to draw definite conclusions. Moreover, the programs are under constant development, so that the currently available version may not be the same as the one that had been evaluated just 2 or 3 years before. Application of the programs also varies; thus, some are being used as universal strategies—that is, they are required of all students—whereas in other cases the intervention only is used for students who have been mandated to take the course in light of their problematic drinking. Nevertheless, the evidence suggests that online approaches can reduce alcohol consumption and possibly also alcohol- related harms. In the evaluation by Hustad and colleagues (2010), incoming freshmen students at a small private university were randomly assigned to one of the intervention programs or to an assessment-only condition. The study found that both programs reduced several measures of student alcohol consumption at a 1-month followup (Hustad et al. 2010). Thus, this type of interventions shows promise and is likely to improve with further development. Among the issues to be considered are whether such programs work equally well for all types of students and at what time the students should be exposed to them. In any case, given the low marginal cost of delivering these programs, demand for them is likely to be high.

## Summary

Significant progress has been made over the past decade with respect to research on college student drinking and the prevention of alcohol-related problems among this population. This especially is true of the Tier 2 interventions that at the time of the NIAAA Task Force had not been evaluated in college settings. Although the research cited in this article comprises only a handful of studies, they clearly establish that community interventions can and do reduce alcohol consumption and subsequent problems in campus communities as they have in the general population. Replication studies certainly would further enhance confidence in the efficacy of these approaches.

However, at least three key general questions remain. First, what is the optimum combination of environmental strategies that will have the greatest impact with the given resources available to a college or university? Second, what is the most efficient way to implement community-level strategies? And third, what would it take for universities to adopt this kind of intervention? All of these questions are related, of course, because adoption of any intervention likely would be driven by the size of the impact and the cost of implementation. These questions already are at the heart of recent individual-level (Tier 1) interventions. And although it may take some time and clever designs to address them at the population level, the hurdles are not insurmountable, especially if college administrators are willing to partner with researchers as they have in the research summarized above.

The common goal for researchers and college professionals alike is to identify the most effective blend of screening, treatment, and individual-, group-, campus-, and community-level prevention components, and to find ways to exploit the synergy that can be expected to result from a coherent set of alcohol programs and policies. The United States has some of the world’s most illustrious institutions of higher education. Therefore, it does not seem out of place to ask some of these colleges and universities to be leaders in developing and implementing comprehensive alcohol prevention strategies and serve as models for communities far beyond their own backyards.
